# Combined Effect of Cold Atmospheric Plasma and Hydrogen Peroxide Treatment on Mature *Listeria monocytogenes* and *Salmonella* Typhimurium Biofilms

**DOI:** 10.3389/fmicb.2019.02674

**Published:** 2019-11-20

**Authors:** Marlies Govaert, Cindy Smet, Davy Verheyen, James L. Walsh, Jan F. M. Van Impe

**Affiliations:** ^1^Flemish Cluster Predictive Microbiology in Foods (CPMF^2^), Ghent, Belgium; ^2^Optimization in Engineering Center-of-Excellence (OPTEC), KU Leuven, Ghent, Belgium; ^3^Chemical & Biochemical Process Technology & Control (BioTeC), Department of Chemical Engineering, KU Leuven, Ghent, Belgium; ^4^Department of Electrical Engineering & Electronics, University of Liverpool, Liverpool, United Kingdom

**Keywords:** cold atmospheric plasma, hydrogen peroxide, inactivation, antimicrobial activity, biofilm, synergy, *Listeria monocytogenes*, *Salmonella* Typhimurium

## Abstract

Cold Atmospheric Plasma (CAP) is a promising novel method for biofilm inactivation as log-reduction values up to 4.0 log_10_ (CFU/cm^2^) have been reported. Nevertheless, as the efficacy of CAP itself is not sufficient for complete inactivation of mature biofilms, the hurdle technology could be applied in order to obtain higher combined efficacies. In this study, CAP treatment was combined with a mild hydrogen peroxide (H_2_O_2_) treatment for disinfection of 1 and 7 day(s) old *Listeria monocytogenes* and *Salmonella* Typhimurium biofilms. Three different treatment sequences were investigated in order to determine the most effective treatment sequence, i.e., (i) first CAP, then H_2_O_2_, (ii) first H_2_O_2_, then CAP, and (iii) a simultaneous treatment of CAP and H_2_O_2_. Removal of the biofilm, induction of sub-lethal injury, and H_2_O_2_ breakdown due to the presence of catalase within the biofilms were investigated in order to comment on their possible contribution to the combined inactivation efficacy. Results indicated that the preferred treatment sequence was dependent on the biofilm forming species, biofilm age, and applied H_2_O_2_ concentration [0.05 or 0.20% (v/v)]. At the lowest H_2_O_2_ concentration, the highest log-reductions were generally observed if the CAP treatment was preceded by the H_2_O_2_ treatment, while at the highest H_2_O_2_ concentration, the opposite sequence (first CAP, then H_2_O_2_) proved to be more effective. Induction of sub-lethal injury contributed to the combined bactericidal effect, while the presence of catalase within the biofilms resulted in an increased resistance. In addition, high log-reductions were partially the result of biofilm removal. The highest overall log-reductions [i.e., up to 5.42 ± 0.33 log_10_ (CFU/cm^2^)] were obtained at the highest H_2_O_2_ concentration if CAP treatment was followed by H_2_O_2_ treatment. As this resulted in almost complete inactivation of the *L. monocytogenes* and *S.* Typhimurium biofilms, the combined treatment of CAP and H_2_O_2_ proved to be a promising method for disinfection of abiotic surfaces.

## Introduction

Due to an increased awareness of the existence of biofilms and their associated problems, the number of biofilm studies has increased significantly. Biofilms can be defined as functional consortia of cells, which are attached to a surface and protected by a self-produced matrix of Extracellular Polymeric Substances (EPS) (Bakke et al., 1984; Costerton et al., 1987; Garrett et al., 2008; Giaouris et al., 2014). They can cause health-related problems, such as foodborne illnesses and chronical infections, as well as economic losses due to food recalls, equipment downtime, and equipment failure (Kumar and Anand, 1998; Garrett et al., 2008; Barry and Kanematsu, 2015). Although many industries are affected by biofilms, the food industry is of particular concern as pathogenic biofilms developed on food contact surfaces can result in foodborne illnesses following consumption of (cross)contaminated food products (Giaouris et al., 2014). *Listeria monocytogenes* and *Salmonella* Typhimurium are two of the most important biofilm forming pathogenic species since these microorganisms can cause listeriosis and salmonellosis, respectively. Within the European Union, listeriosis has a relatively low notification rate (0.48/100,000 capita), but a very high fatality rate of 13.8%. Salmonellosis on the other hand, has a high notification rate (19.7/100,000 capita) and a fatality rate of 0.25% (EFSA and ECDC, 2018).

Since traditional inactivation technologies [i.e., rinsing with (hot) water and antimicrobial agents] have been reported to be insufficient for biofilm inactivation, novel technologies should be investigated (Costerton et al., 1987; Kumar and Anand, 1998; Jefferson, 2004; Giaouris et al., 2014). Cold Atmospheric Plasma (CAP) is one of those promising inactivation technologies. Plasma can be defined as a partially or wholly ionized gas (consisting of ions, photons, free electrons, and activated neutral species), which can be created by addition of energy to a gas (Tendero et al., 2006; Banu et al., 2012; Fernández and Thompson, 2012). The gas can be energized by means of an electric discharge at room temperature and at atmospheric pressure, resulting in the creation of a specific plasma type, i.e., CAP. This novel inactivation technology has some important advantages, i.e., (i) it is fast, (ii) it can be created at a low temperature and low pressure, (iii) plasma components fade out immediately after treatment, and (iv) cells are inactivated by a variety of mechanisms (Misra et al., 2011; Banu et al., 2012; Fernández and Thompson, 2012; Patil et al., 2016). The complete inactivation mechanism of CAP is not fully understood yet, however, three main factors have been reported in literature to contribute to its bactericidal effect. These factors are UV radiation, charged particles, and reactive oxygen and nitrogen species (ROS and RNS) (Laroussi, 2005; Surowsky et al., 2015; Bourke et al., 2017). Dependent on the specifically involved species, their interaction with the (biofilm-associated) cells can result in (i) a damaged (outer) cell membrane, (ii) an altered structure and/or altered functional properties of the macromolecules, and (iii) a negative influence on the DNA (Misra et al., 2011; Fernández and Thompson, 2012; Puligundla and Mok, 2017).

Although in previous research log-reductions up to 4 log_10_ have been obtained following CAP treatment of *L. monocytogenes* and *S.* Typhimurium biofilms, additional research is still required to obtain higher log-reductions and, consequently, further reduce the risk of (cross)contamination of food products (Ziuzina et al., 2015; Govaert et al., 2019). A possible solution to obtain these higher log-reductions or, ideally, a complete inactivation, could be the combination of different inactivation strategies. Within the food industry, the hurdle principle has been used excessively to ensure food safety. By combining different mild (synergistic) treatments, food safety can be guaranteed while still ensuring the quality of the food product (Leistner, 2000). The hurdle principle could be applied as well for inactivation of biofilms growing on abiotic surfaces, i.e., CAP treatment could be combined with a mild (chemical) treatment to obtain higher log-reductions.

Hydrogen peroxide (H_2_O_2_) is often used for disinfection of abiotic food contact surfaces, either on its own or in combination with other chemicals. The antibacterial effect of this chemical involves damage to DNA, proteins, lipids, and cell membranes, eventually resulting in microbial inactivation and/or destruction of the biofilm matrix (Bell et al., 1997; Ukuku et al., 2012). H_2_O_2_ is generally recognized as safe (GRAS) and its application for food products has been approved by the US Food and Drug Administration (Ukuku et al., 2012). With respect to its efficacy for biofilm inactivation, the studies of Christensen et al. (1990) and Shikongo-Nambabi et al. (2010) reported that H_2_O_2_ was capable of (partially) inactivating mature marine and *Vibrio alginolyticus* biofilms, respectively. However, contradictory findings have been reported as well, i.e., H_2_O_2_ was for example not capable to inactivate mature *Pseudomonas aeruginosa* biofilms due to the presence of catalase within these specific biofilms (Stewart et al., 2000).

Although H_2_O_2_ exhibits a biofilm inactivating potential which is in many cases insufficient to ensure food safety (Stewart et al., 2000; Shikongo-Nambabi et al., 2010), this commonly used antimicrobial agent could still be combined with CAP to enhance CAP’s promising antimicrobial activity against pathogenic biofilms. As H_2_O_2_ treatment results in a destruction of the biofilm matrix (Bell et al., 1997; Ukuku et al., 2012), a higher penetration capacity of the plasma species and an increased overall inactivation could be obtained. In addition, as sub-lethal injury of biofilm-associated cells following CAP treatment has been reported (Govaert et al., 2019), these sub-lethally CAP injured cells might possibly become more susceptible to a subsequent treatment with H_2_O_2_, again probably increasing the overall inactivation. A similar trend was for example observed within the research of Smet et al. (2017), where CAP treated food model systems containing *L. monocytogenes* and *S.* Typhimurium cells had a prolonged shelf life when these samples were, in addition to the CAP treatment, subjected to high levels of salt. However, the opposite trend has been observed as well, i.e., (sub-lethally) injured cells became more resistant as a consequence of stress hardening following exposure to different sub-lethal stress factors such as sub-lethal temperatures (Wesche et al., 2009). Therefore, it is of high importance to investigate the influence of this phenomenon on the overall efficacy of a combined CAP and H_2_O_2_ treatment.

The main goal of this research was to investigate the combined efficacy of CAP and H_2_O_2_ for inactivation of 1 and 7 day(s) old *L. monocytogenes* (Gram positive) and *S.* Typhimurium (Gram negative) biofilms. Initially, the efficacy of an individual H_2_O_2_ treatment [10 or 30 min – 0.05 or 0.20% (v/v)] was investigated and compared with the antimicrobial activity of an individual CAP treatment (10 min). Next, the efficacy of a combined CAP (10 min) and H_2_O_2_ [10 min – 0.05 or 0.20% (v/v)] treatment was determined while applying three different treatment sequences, i.e., biofilms were (i) first treated with CAP, followed by H_2_O_2_, (ii) first treated with H_2_O_2_, followed by CAP, or (iii) simultaneously treated with CAP and H_2_O_2_. Possible increased combined treatment efficacies were examined by comparing the combined log-reductions with the sum of the log-reductions obtained following the individual CAP and H_2_O_2_ treatment. For each of the individual treatments, the total biofilm mass and the percentage of sub-lethally injured cells were determined before and after the treatment in order to comment on a possible contribution of biofilm removal and/or induction of sub-lethal injury to the (increased) efficacy of the combined treatments. Finally, the presence of catalase within the different model biofilms was determined to possibly explain their (lack of) resistance toward H_2_O_2_ treatment.

## MATERIALS AND METHODS

### Experimental Design

**FIGURE 1:**
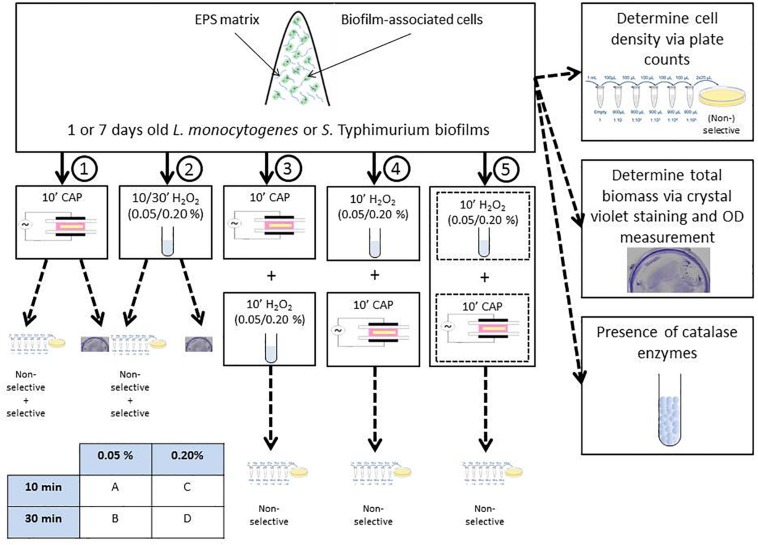
Experimental design used within the presented research.

Within this study, the experimental design illustrated in Figure 1 was used. Initially, 1 and 7 day(s) old control *L. monocytogenes* and *S.* Typhimurium biofilms were prepared and their cell density and total biofilm mass were determined via plate counts (on non-selective and selective media) and crystal violet staining, respectively. Next, 1 and 7 day(s) old biofilms were treated with five different treatment conditions. Treatment condition (1) involved an individual CAP treatment of 10 min, while treatment condition (2) concerned an individual H_2_O_2_ treatment. For the latter treatment, two different H_2_O_2_ concentrations and two different contact times were investigated, i.e., 0.05 or 0.20% (v/v) and 10 or 30 min. Therefore, treatment condition (2) has been further subdivided in (2A), (2B), (2C), and (2D). For the combined treatment conditions, 1 and 7 day(s) old model biofilms were exposed to (3) 10 min of CAP treatment followed by 10 min of H_2_O_2_ treatment [0.05/0.20% (v/v)], (4) 10 min of H_2_O_2_ treatment [0.05/0.20% (v/v)] followed by 10 min of CAP treatment, and (5) a simultaneous treatment of CAP and H_2_0_2_ [0.05/0.20% (v/v)] for a duration of 10 min. For these combined treatments, a similar subdivision as for treatment condition (2) has been applied to indicate the precise H_2_O_2_ concentration and contact time. For all CAP treatments, optimal biofilm inactivation conditions were selected as determined in the research of Govaert et al. (2019). This optimal set of conditions involved the use of a Dielectric Barrier Discharge (DBD) electrode, helium as feed gas, and an input voltage of 21.88 V. Following each treatment, viable plate counts (on non-selective medium) were used to determine the remaining cell density of the biofilms (see section “Biofilm Quantification via Viable Plate Counts” for a more detailed explanation). Consequently, it was possible to (i) determine the (total) log-reductions obtained with all five (combined) treatments and (ii) comment on a possible increased efficacy of the combined CAP and H_2_O_2_ treatment. For the individual treatment conditions (1) and (2), selective media were used as well in order to determine the percentage of sub-lethally injured cells and their possible contribution to the (increased) overall efficacy of the tested combined treatments. To investigate if the (combined) log-reductions were solely the result of biofilm inactivation or also (partially) due to biofilm removal, the total biofilm mass was also determined after biofilm treatment with treatment conditions (1) and (2). Finally, the presence of catalase within the different model biofilms was determined in order to comment on a possible contribution of these enzymes to the resistance of the biofilms toward H_2_O_2_ treatment.

It should be mentioned that throughout this manuscript, the term “inactivation” was used to indicate a significant reduction of the biofilm cell density, not necessarily implying that all biofilm-associated cells were killed following the different treatment conditions.

### Microorganisms and Pre-culture Conditions

*Listeria monocytogenes* LMG 23775 (isolated from sausages) and *S.* Typhimurium LMG 14933 (isolated from bovine liver) were used within this study. These strains were both acquired from the Belgian Co-ordinated Collections of Micro-organisms/Laboratory of Microbiology (BCCM/LMG) of Ghent University in Belgium. Stock-cultures were stored at −80°C in Brain Heart Infusion broth (BHI, VWR International, Belgium) and Tryptic Soy Broth (TSB, Becton Dickinson, United States), respectively, which were both supplemented with 20% (v/v) glycerol (VWR International, Belgium).

For every experiment, a purity plate was prepared by spreading a loopful of stock-culture onto an agar plate [Lennox LB broth (Becton Dickinson, United States) supplemented with 14 g/L bacteriological agar (VWR Chemicals, Belgium) and 5 g/L NaCl (Sigma-Aldrich, United States)]. Agar plates were incubated for 24 h at 30 (*L. monocytogenes*) or 37°C (*S.* Typhimurium), which are the optimal growth temperatures for these microorganisms (BCCM, 2017). Pre-cultures were prepared by transferring one colony from the incubated purity plate into an Erlenmeyer flask containing 20 mL of broth (Lennox LB broth supplemented with 5 g/L NaCl). Pre-cultures were again incubated for 24 h at previously mentioned optimal growth temperatures to obtain stationary phase cultures with a cell density of approximately 10^9^ CFU/mL.

### Model Biofilm Development

The detailed biofilm formation protocol has been presented before in the research of Govaert et al. (2018b). Briefly, small polystyrene Petri dishes (50 mm diameter, 9 mm height, Simport, Canada) were used as abiotic surfaces. Each Petri dish was inoculated with 1.2 mL of inoculum, which was prepared by making a 100-fold dilution of the stationary phase pre-culture. BHI and 20-fold diluted TSB (TSB/20) were used as dilution medium for *L. monocytogenes* and *S.* Typhimurium, respectively. After inoculation, Petri dishes were closed and gently shaken to make sure the inoculum covered the entire surface. Finally, Petri dishes were incubated for 1 or 7 day(s) at 30 (*L. monocytogenes*) or 25°C (*S.* Typhimurium). The 1 day old biofilms were the reference biofilms used within the study of Govaert et al. (2019) to determine the most optimal CAP treatment conditions for biofilms, while in the study of Govaert et al. (2018a), the 7 days old biofilms proved to have to highest resistance toward CAP treatment. Therefore, the same biofilm ages were used within the presented study.

### CAP Equipment, Hydrogen Peroxide Solution Preparation, and Inactivation Procedures

For all treatment conditions involving CAP, biofilms were treated for 10 min while applying the optimal inactivation conditions determined in the research of Govaert et al. (2019). A Dielectric Barrier Discharge (DBD) electrode was used to energize a helium feed gas (4 L/min; purity 99.996%) at an input voltage of 21.88 V, which resulted in an output voltage and a dissipated power value of approximately 6.5 kV and 7.0 W, respectively. More detailed specifications of the CAP equipment can be found in the previously mentioned study. For all treatment conditions involving H_2_O_2_, a 35% (v/v) stock solution (VWR Chemicals, Belgium) was diluted with sterile demineralized water to obtain the appropriate working concentrations of 0.05 and 0.20% (v/v), which were selected based on the study of Shikongo-Nambabi et al. (2010).

Prior to each individual or combined treatment, the 1 or 7 day(s) old biofilms were rinsed three times with 1.2 mL of sterile Phosphate Buffered Saline (PBS) solution (to remove the remaining weakly attached/planktonic cells) and allowed to dry. For the individual CAP treatment [i.e., condition (1)], a single model biofilm was placed inside the electrode chamber. Prior to the CAP treatment of 10 min, this enclosure was flushed with helium for 4 min to obtain a homogeneous environment. After the CAP treatment, the biofilms were immediately quantified via viable plate counts or crystal violet staining. For the individual H_2_O_2_ treatment [i.e., conditions (2A)–(2D)], 1.2 mL of the appropriate H_2_O_2_ solution was added to the Petri dishes and a contact time of 10 or 30 min was applied. Afterward, the H_2_O_2_ solution was removed with a pipette, the biofilms were again rinsed three times with 1.2 mL of sterile PBS solution to remove the active solution, and the biofilms were allowed to dry prior to quantification. For the CAP treatment part involved within the combined treatment procedures, biofilms were always treated for 10 min while using the optimal inactivation conditions previously discussed. With respect to the H_2_O_2_ treatment part, the applied concentration [i.e., 0.05 and 0.20% (v/v)] and treatment duration (i.e., 10 min) were selected based on the results obtained for the individual H_2_O_2_ treatment [i.e., conditions (2A)–(2D)] and the treatment procedure was similar to the one previously described. For treatment condition (5), only one H_2_O_2_ treatment duration was possible (i.e., 10 min) since this chemical treatment had to be performed simultaneously with the CAP treatment. However, it should be mentioned that the H_2_O_2_ treatment had in reality a duration of approximately 15 min due to the installation/removal of the sample in/from the electrode chamber and the 4 min during flushing time required to obtain a homogenous environment within this electrode chamber (Govaert et al., 2019).

Finally, preliminary tests have been performed to assure the efficacy of PBS as rinsing agent for removal of the oxidative H_2_O_2_ solution. For these experiments, the 1 day old model biofilms were subjected to a H_2_O_2_ treatment using the highest concentration [0.20% (v/v)] and a contact time of 10 min. A colorimetric assay (see section “Remaining Percentage of H_2_O_2_ Following Contact of the H_2_O_2_ Solutions With the Model Biofilms”) was used to determine the remaining H_2_O_2_ concentration of the final rinsing solution. These preliminary tests proved that the H_2_O_2_ concentration of this solution was below the detection limit. Therefore, it was concluded that PBS was sufficiently effective to remove the oxidative H_2_O_2_ solution.

### Biofilm Quantification via Viable Plate Counts

The cell density of the untreated and treated biofilms was determined via viable plate counts. Therefore, 2 mL of sterile PBS solution was added to the (rinsed, dried, and, if applicable, treated) biofilms and a cell scraper (blade width 20 mm, Carl Roth GmbH + Co., Germany) was used to remove the biofilm from the surface. Afterward, the obtained cell suspension was transferred to an empty micro centrifuge tube of 1.5 mL and vortexed to disperse cellular aggregates. Serial decimal dilutions of the vortexed cell suspension were prepared [in 0.85% (w/v) NaCl solution] and plated on agar plates. For each of the dilutions, three drops of 20 μL were plated on non-selective and, if applicable, selective media (Miles et al., 1938). Brain Heart Infusion Agar (BHIA, BHI supplemented with 14 g/L bacteriological agar) and PALCAM (VWR Chemicals, Belgium) were used as non-selective and selective medium for *L. monocytogenes*, while Tryptic Soy Agar (TSA, TSB supplemented with 14 g/L bacteriological agar) and Xylose Lysine Deoxycholate Agar (XLDA, Merck & Co., United States) were used for *S.* Typhimurium. Before counting the colonies, agar plates were incubated for (at least) 24 h at 30 (BHIA/PALCAM) or 37°C (TSA/XLDA).

The cell density was considered to be below the detection limit when the number of CFUs in the three droplets of 20 μL (= 60 μL in total) was below 10. In that case, the detection limit [i.e., 1.2 log(CFU/cm^2^)] was used to calculate the log-reduction values obtained at these specific treatment conditions.

### Biofilm Quantification via Crystal Violet Staining

The total biofilm mass of the untreated and treated biofilms [with individual treatment conditions (1) and (2A)–(2D)] was determined using the crystal violet assay as explained in Govaert et al. (2018b). In summary, the different steps of this assay were: (i) rinsing of the biofilms with PBS, (ii) fixation of adhering biofilms with methanol (99% (v/v), VWR Chemicals, Belgium), (iii) staining with a 2% (v/v) crystal violet solution (Sigma-Aldrich, United States), (iv) removal of excess stain, (v) addition of glacial acetic acid solution (33% (v/v), VWR International, Belgium) to re-dissolve the remaining stain, and (vi) optical density (OD) measurement at 570 nm [VersaMax tunable microplate reader (Molecular devices, United Kingdom)]. If the OD was higher than one, the solution was diluted using the glacial acetic acid solution and a correction factor was incorporated to determine the OD of the original solution.

### Remaining Percentage of H_2_O_2_ Following Contact of the H_2_O_2_ Solutions With the Model Biofilms

The research of Stewart et al. (2000) reported that H_2_O_2_ was not capable to inactivate mature *P. aeruginosa* biofilms due to the presence of catalase within this particular biofilm. Therefore, the presence of catalase within the model biofilms developed by the catalase positive *L. monocytogenes* and *S.* Typhimurium cells was examined in order to explain their (lack of) resistance toward H_2_O_2_ treatment.

A colorimetric method involving the use of Titanium Oxysulfate (TiOSO_4_) (Lu et al., 2017) was used to determine the percentage of the original H_2_O_2_ concentration that still remained following contact of the 0.05 and 0.20% (v/v) H_2_O_2_ solutions with the 1 and 7 day(s) old *L. monocytogenes* and *S.* Typhimurium biofilms. First, the absorbance of the original H_2_O_2_ solutions [i.e., 0.05 and 0.20% (v/v)] was determined by transferring 100 μL of the solution to a single well of a 96-well microtiter plate (Greiner Bio-One, Austria) containing 10 μL of TiOSO_4_ (Sigma-Aldrich, United States). This mixture was incubated for 10 min at room temperature and in the dark. Following this incubation period, a microplate reader (VersaMax tunable microplate reader, Molecular devices, United Kingdom) was used to measure the absorbance at 405 nm. After this, the (rinsed and dried) 1 and 7 day(s) old biofilms were subjected to treatment conditions (2A) and (2B), i.e., 1.2 mL of the 0.05 or 0.20% (v/v) H_2_O_2_ solution was added to each of the model biofilms and a contact time of 10 min was applied. Afterward, the biofilms were removed from the surface using a cell scraper and the obtained H_2_O_2_ cell suspensions were filtered-sterilized (0.2 μM pore size, Sarstedt, Germany). To determine the absorbance of these filter-sterilized H_2_O_2_ solutions, the previously explained colorimetric assay was applied. Finally, for each of the model biofilms and for both examined H_2_O_2_ concentrations, the remaining percentage of H_2_O_2_ was calculated using Equation 1.

(1) $$\text{Remaining}\,\,{\text{H}}_{2}{\text{O}}_{2}\mathrm{percentage}\left(\%\right)=\frac{A\,b\,s\,o\,r\,b\,a\,n\,c\,e\,\,\,\,a\,f\,t\,e\,r\,\,\,\,\,c\,o\,n\,t\,a\,c\,t\,\,\,\,w\,i\,t\,h\,\,\,\,\mathrm{mod}\,e\,l\,\,\,b\,i\,o\,f\,i\,l\,m}{A\,b\,o\,r\,b\,a\,n\,c\,e\,c\,o\,r\,r\,e\,s\,p\,o\,n\,d\,i\,n\,g\,o\,r\,i\,g\,i\,n\,a\,l\,{\text{H}}_{2}\,{\text{O}}_{2}\,s\,o\,l\,u\,t\,i\,o\,n}$$

A low remaining percentage of the original H_2_O_2_ concentration was deemed to be a consequence of the presence of catalase within the model biofilms. This low remaining percentage can explain the possible resistance of the model biofilms toward H_2_O_2_ treatment and/or the lack of an increased efficacy of the combined CAP and the H_2_O_2_ treatment.

### Estimation of the Percentage of Sub-Lethally Injured Cells

To calculate the percentage of sub-lethal injury (% SI) following each individual treatment condition [i.e., conditions (1) and (2)], the equation of Busch and Donnelly (1992) (Equation 2) was used.

(2) $$\%\mathrm{SI}=\frac{\mathrm{CFU}\,\mathrm{non}-\mathrm{selective}\,\mathrm{medium}-\mathrm{CFU}\,\mathrm{selective}\,\mathrm{medium}}{\mathrm{CFU}\,\mathrm{non}-\mathrm{selective}\,\mathrm{medium}}\times 100$$

Values were considered as zero in case negative values were obtained as a consequence of (small) plating errors specific to the applied enumeration method.

Calculated sub-lethal injury percentages of the 1 and 7 day(s) old untreated *L. monocytogenes* and *S.* Typhimurium biofilms were compared with those obtained following each of the corresponding individual treatment conditions. A significant increase in sub-lethal injury was considered to be an important factor possibly stimulating an improved efficacy of any subsequent treatment.

### Assessment of the Efficacy of the Combined Treatments

In order to comment on the efficacy of the combined CAP and H_2_O_2_ treatments for inactivation of biofilms, the total log-reductions (on non-selective medium)] obtained following treatment conditions (3), (4), and (5) were compared with the sum of individual treatments (SIT). To calculate the SIT values, the log-reductions (on non-selective medium) obtained with treatment conditions (1) and (2) were added up. Two different SIT values were defined, i.e., one for each H_2_O_2_ concentration [(2A) and (2C)]. When the log-reductions of the combined treatments were significantly higher than their corresponding SIT value, the combined treatment was considered to have an increased combined treatment efficacy. In contrary, when these combined log-reductions were significantly lower, the applied treatments were considered to result in a decreased combined treatment efficacy. Since treatment conditions (3)–(5) involved three different treatment sequences, it was possible to comment on the preferred treatment sequence to obtain the highest possible log-reductions.

### Statistical Analysis

Analysis of variance (ANOVA) tests were performed to determine whether there were any significant differences between (i) the obtained log-reductions following each of the individual treatments, (ii) the combined log-reductions and their corresponding SIT value, (iii) the initial percentages of sub-lethal injury (untreated biofilms) and those obtained following each of the individual treatment conditions, (iv) the OD of the biofilms measured before and after the individual treatment conditions, and (v) the remaining percentages of H_2_O_2_ obtained following contact with the different model biofilms. A confidence level of 95.0% (α = 0.05) was applied and Fisher’s Least Significant Difference (LSD) test was used to distinguish which values were significantly different from others. The analyses were performed using Statgraphics 18 software (Statistical Graphics, Washington, DC, United States) and significant differences were indicated with different uppercase or lowercase letters (e.g., “A” or “a”) or numbers (e.g., “I” or “i”).

## Results

### Efficacy of an Individual CAP and an Individual H_2_O_2_ Treatment for Biofilm Inactivation

**FIGURE 2:**
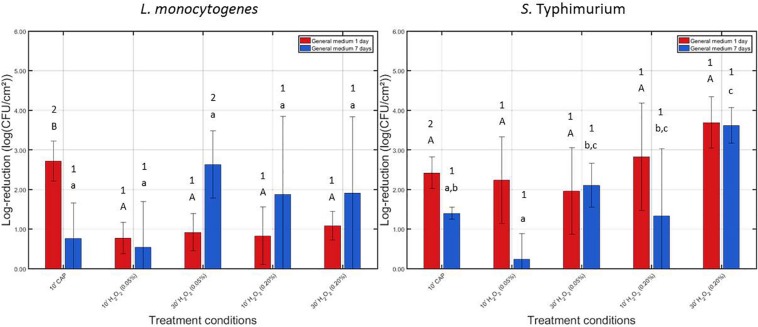
Log-reductions obtained following treatment of the 1 and 7 days old *L. monocytogenes* and *S.* Typhimurium biofilms with individual treatment conditions (1) (i.e., 10 min CAP treatment) and (2) [i.e., 10 or 30 treatment with a 0.05 or 0.20% (v/v) H_2_O_2_ solution]. For each condition, three independent replicates have been performed. Average values and corresponding standard deviations have been calculated and presented in the figure. Significant differences (*p* ≤ 0.05) obtained following the different individual treatment procedures are indicated with a different uppercase (1 day old) or lowercase (7 days old) letter, with “A” or “a” bearing the lowest value. To indicate the influence of the biofilm age on the efficacy of these individual treatment procedures, different numbers were applied, with “1” bearing the lowest value.

In Figure 2, the log-reductions obtained following (i) 10 min of CAP treatment and (ii) 10 or 30 min of H_2_O_2_ treatment with a 0.05 or 0.20% (v/v) H_2_O_2_ solution can be observed. Results have been included for both biofilm forming species and both biofilm ages. The results obtained following 10 min of CAP treatment (for both species and both biofilm ages) were previously presented in the studies of Govaert et al. (2018a, 2019). The initial cell densities of the 1 and 7 day(s) old model biofilms were used to calculate the log-reduction values obtained following each individual treatment. For *L. monocytogenes*, these values were 7.16 ± 0.33 and 6.35 ± 0.72 log_10_ (CFU/cm^2^), respectively. For *S.* Typhimurium, on the other hand, the initial cell densities were 6.48 ± 0.27 and 5.79 ± 0.26 log_10_ (CFU/cm^2^), respectively.

For the 1 day old *L. monocytogenes* biofilm, it can be observed that CAP treatment was significantly more effective (*p* ≤ 0.05) than H_2_O_2_ treatment since the former treatment condition resulted in a log-reduction of 2.72 ± 0.51 log_10_ (CFU/cm^2^), while the latter treatments only resulted in log-reductions ranging between 0.78 and 1.09 log_10_ (CFU/cm^2^). In addition, for this biofilm age, the concentration and treatment time did not influence (*p* > 0.05) the efficacy of the H_2_O_2_ treatment, i.e., similar log-reductions were obtained independently from aforementioned treatment characteristics. For the 7 days old *L. monocytogenes* biofilm, CAP treatment was less effective (*p* ≤ 0.05) than for the 1 day old biofilm, i.e., obtained log-reductions decreased from 2.72 ± 0.51 to 0.77 ± 0.89 log_10_ (CFU/cm^2^). In addition, as for the 1 day old *L. monocytogenes* biofilm, the efficacy of the H_2_O_2_ treatment was again independent (*p* > 0.05) of the applied treatment characteristics. However, the latter observation might be a consequence of the high variability of the log-reductions obtained following treatment of the 7 days old biofilms.

For the 1 day old *S.* Typhimurium biofilm, no significant differences (*p* > 0.05) can be observed between the log-reductions obtained following CAP treatment and those obtained following H_2_O_2_ treatment. Both treatments were equally effective (*p* > 0.05) and resulted in log-reductions ranging between 1.96 ± 1.09 and 3.70 ± 0.65 log_10_ (CFU/cm^2^). In addition, as for *L. monocytogenes*, the efficacy of the H_2_O_2_ treatment was independent (*p* > 0.05) of the applied treatment characteristics. If the average values are compared, an increased antimicrobial activity can be obtained with an increased H_2_O_2_ concentration. For the 7 days old *S.* Typhimurium biofilm, it can be observed that the biofilm-associated cells were less susceptible (*p* ≤ 0.05) to CAP treatment compared to the corresponding 1 day old biofilm. For the highest H_2_O_2_ concentration [i.e., 0.20% (v/v)] and the longest treatment time (i.e., 30 min), H_2_O_2_ treatment became more effective (*p* ≤ 0.05) than CAP since the former treatment resulted in a log-reduction of 3.62 ± 0.45 log_10_ (CFU/cm^2^), while the latter treatment only resulted in a log-reduction of 1.40 ± 0.15 log_10_ (CFU/cm^2^). Consequently, for this type of model biofilm, the efficacy of the H_2_O_2_ treatment was dependent on the applied treatment characteristics.

Comparing the efficacy of the individual treatments for inactivation of both biofilm forming species, it can be observed that these both have a similar resistance toward CAP treatment. However, the *L. monocytogenes* biofilm-associated cells appeared to be less susceptible to H_2_O_2_ treatment than those present in the *S.* Typhimurium biofilms.

Based on the results observed in Figure 2, it can be concluded that the biofilm inactivation efficacy of H_2_O_2_ is in general independent of the applied treatment time, i.e., only for the 7 days old *S.* Typhimurium biofilm, an increased treatment time seems to result in increased log-reductions. Therefore, it was decided to investigate possible synergetic effects between CAP and H_2_O_2_ for both tested H_2_O_2_ concentrations, but only for the shortest treatment time (i.e., 10 min). Consequently, for future real-life applications, the equipment downtime could be kept as low as possible while (potentially) obtaining high log-reductions.

### Efficacy of the Combined CAP and H_2_O_2_ Treatments for Biofilm Inactivation

**FIGURE 3:**
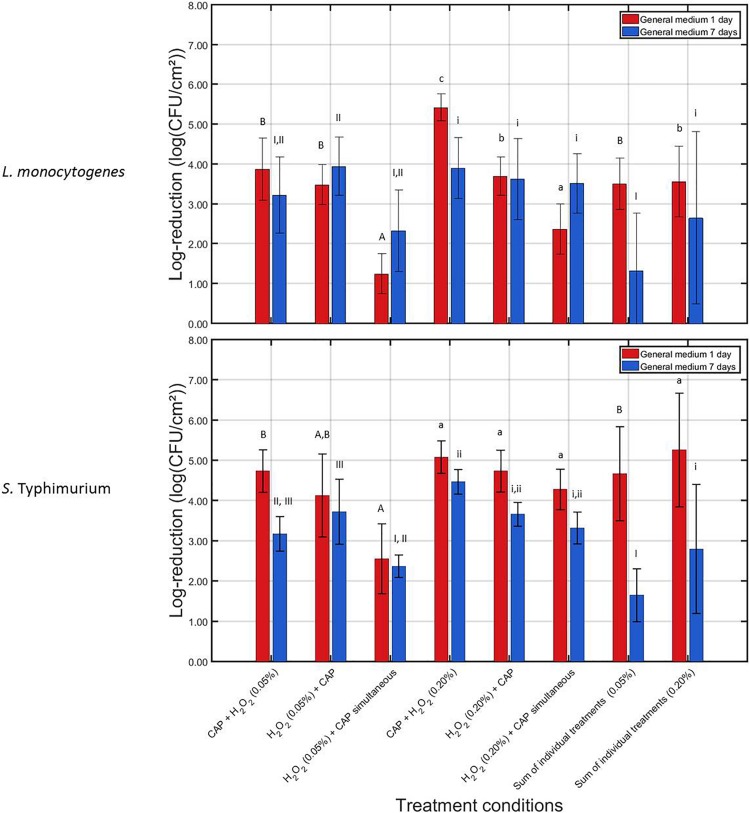
Log-reductions obtained following treatment of the 1 and 7 days old *L. monocytogenes* and *S.* Typhimurium biofilms with combined treatment conditions (3) {i.e., 10 min CAP followed by 10 min H_2_O_2_ [0.05 or 0.20% (v/v)]}, (4) {i.e., 10 min H_2_O_2_ [0.05 or 0.20% (v/v)]} followed by 10 min CAP), and (5) {i.e., a 10 min during simultaneous CAP and H_2_O_2_ [0.05 or 0.20% (v/v)] treatment}. For each condition, three independent replicates have been performed. Average values and corresponding standard deviations have been calculated and presented in the figure. The sum of individual treatments (SIT) values have been calculated for each H_2_O_2_ concentration and each biofilm age and were compared with the log-reductions obtained for their corresponding combined treatments. Significant (*p* = 0.05) differences have been indicated by means of uppercase letters [1 day old – 0.05% (v/v)], lowercase letters [1 day old – 0.20% (v/v)], uppercase numbers [7 days old – 0.05% (v/v)], or lowercase numbers [7 days old – 0.20% (v/v)], with “A,” “a,” “I,” or “i” bearing the lowest value.

In Figure 3, the log-reductions obtained following the combined CAP and H_2_O_2_ treatments can be observed for the 1 and 7 days old *L. monocytogenes* and *S.* Typhimurium biofilms. In addition, for both biofilm ages, the corresponding SIT values were presented.

For the 1 day old *L. monocytogenes* biofilm, it can be observed that the combined log-reductions involving the lowest H_2_O_2_ concentration [i.e., 0.05% (v/v)] ranged between 1.25 ± 0.50 and 3.88 ± 0.78 log_10_ (CFU/cm^2^), which is still rather low as the initial cell density of the untreated biofilm was 7.16 ± 0.33 log_10_ (CFU/cm^2^). In addition, the combined treatments did not result in an increased combined treatment efficacy (*p* > 0.05). The contrary was even observed, i.e., for the simultaneous treatment, the combined log-reduction was significantly lower (*p* ≤ 0.05) than the corresponding SIT value, indicating a decreased combined treatment efficacy. For the highest H_2_O_2_ concentration [i.e., 0.20% (v/v)], the combined log-reduction values ranged between 2.37 ± 0.48 and 5.42 ± 0.33 log_10_ (CFU/cm^2^), which is much higher than the ones obtained at the lowest H_2_O_2_ concentration. In addition, an increased combined treatment efficacy (*p* ≤ 0.05) can be observed between CAP and H_2_O_2_, however, only when the CAP treatment was followed by the H_2_O_2_ treatment. Applying the opposite sequence (i.e., first H_2_O_2_, then CAP) or the simultaneous treatment resulted in no significant (*p* > 0.05) differences or in a decreased combined treatment efficacy, respectively. For the 7 days old *L. monocytogenes* biofilm, an increased combined treatment efficacy (*p* ≤ 0.05) was only observed while applying the lowest H_2_O_2_ concentration [i.e., 0.05% (v/v)] when the chemical treatment was followed by the CAP treatment. For all other investigated combined treatments (involving both H_2_O_2_ concentrations), no significant (*p* > 0.05) differences were observed between the combined log-reductions and their corresponding SIT value. The obtained log-reduction values ranging between 2.32 ± 1.03 and 3.94 ± 0.73 log_10_ (CFU/cm^2^) were therefore not able to sufficiently reduce the initial cell density of the 7 days old *L. monocytogenes* biofilms.

For the 1 day old *S.* Typhimurium biofilms, only the simultaneous H_2_O_2_ and CAP treatment using the 0.05% (v/v) concentration resulted in significant (*p* ≤ 0.05) differences between the combined log-reduction value and the corresponding SIT value. For this specific treatment condition, a decreased combined treatment efficacy was observed. Nevertheless, despite the lack of an increased combined treatment efficacy, the combined log-reductions ranged between 2.55 ± 0.87 and 5.08 ± 0.41 log_10_ (CFU/cm^2^). This latter number, obtained at the highest H_2_O_2_ concentration when the chemical treatment was preceded by the CAP treatment, is very promising for future application as the cell density of the untreated 1 day old *S.* Typhimurium biofilm was 6.48 ± 0.27 log_10_ (CFU/cm^2^). For the 7 days old *S.* Typhimurium biofilm, an increased combined treatment efficacy (*p* ≤ 0.05) was observed for both non-simultaneous treatments while applying the lowest H_2_O_2_ concentration. While the highest H_2_O_2_ concentration was applied, an increased combined treatment efficacy (*p* ≤ 0.05) could be observed when the CAP treatment was followed by the chemical treatment. At the lowest H_2_O_2_ concentration, the combined log-reductions were, however, limited to values ranging between 2.36 ± 0.28 and 3.72 ± 0.81 log_10_ (CFU/cm^2^), while for the highest H_2_O_2_ concentration, the combined log-reductions reached values up to 4.47 ± 0.31 log_10_ (CFU/cm^2^). Since the 7 days old *S.* Typhimurium biofilms had an initial cell density of 5.79 ± 0.26 log_10_ (CFU/cm^2^), this means that the biofilm cell density can be reduced up until the detection limit.

When comparing the results obtained for both microorganisms and both biofilm ages, it can be observed that the *L. monocytogenes* biofilms were in general more resistant toward the combined treatment, i.e., lower log-reduction values were obtained for this biofilm forming species compared to those obtained for inactivation of the *S.* Typhimurium biofilms. With respect to the biofilm age, it can be observed that the 7 days old biofilms were in general more resistant toward the combined treatments than the 1 day old biofilms.

### Percentage of Sub-Lethal Injury (SI) Following an Individual CAP and an Individual H_2_O_2_ Treatment

**FIGURE 4:**
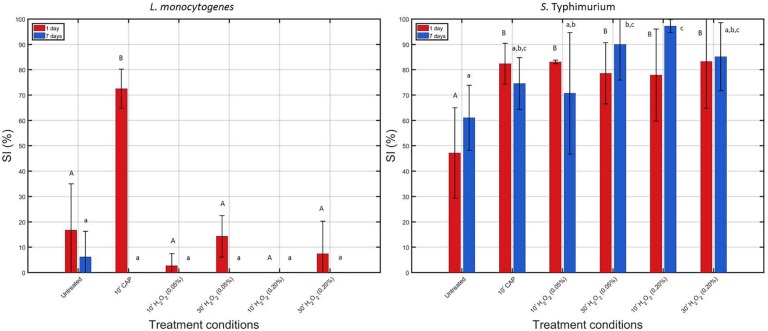
Comparison of the percentage of sub-lethally injured (%SI) cells present in the untreated biofilms and those treated with individual treatment conditions (1) (i.e., 10 min CAP) and (2) [i.e., 10 or 30 min treatment with a 0.05 or 0.20% (v/v) H_2_O_2_ solution]. Results have been included for both biofilm forming species and both biofilm ages. For each condition, three independent replicates have been performed. Average values and corresponding standard deviations have been calculated and presented in the figure. Significant (*p* ≤ 0.05) differences have been indicated with a different uppercase (1 day old) or lowercase (7 days old) letter, with “A” or “a” bearing the lowest value.

In Figure 4, calculated percentages of sub-lethal injury have been presented for each of the individual treatments. As mentioned before, for each biofilm age and each species, the obtained percentages were compared with the initial percentages calculated for the corresponding untreated model biofilm.

When comparing the initial percentages of SI (obtained for the untreated biofilms), it can be observed that SI mainly occurred for the *S.* Typhimurium biofilm-associated cells, which has been reported before in the research of Govaert et al. (2019). In addition, it can in general be concluded that the *S.* Typhimurium biofilm-associated cells appeared to be more susceptible to SI than the *L. monocytogenes* biofilm-associated cells. For the 1 and 7 day(s) old *S*. Typhimurium biofilms, an increase in sub-lethal injury occurred following each of the individual treatments [although not always significantly (*p* > 0.05) for the 7 days old biofilms], while for the *L. monocytogenes* biofilms, a significant (*p* ≤ 0.05) increase in sub-lethal injury was only observed when the 1 day old biofilms were CAP treated for 10 min.

### Determination of the Total Biofilm Mass Following an Individual CAP and an Individual H_2_O_2_ Treatment

**FIGURE 5:**
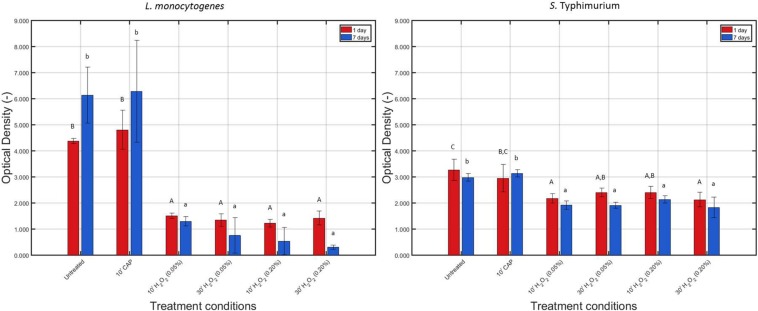
Optical density (OD) values (−) obtained following treatment of the 1 and 7 days old *L. monocytogenes* and *S.* Typhimurium biofilms with individual treatment conditions (1) (i.e., 10 min CAP treatment) and (2) [i.e., 10 or 30 min treatment with a 0.05 or 0.20% (v/v) H_2_O_2_ solution]. For each condition, three independent replicates have been performed. Average values and corresponding standard deviations have been calculated and presented in the figure. Values obtained following treatment of the biofilms were compared with those obtained for their corresponding untreated biofilms. Significant (*p* ≤ 0.05) differences have been indicated with an uppercase (1 day old) or lowercase (7 days old) letter, with “A” or “a” bearing the lowest value.

The measured OD values before and after each individual treatment condition have been presented in Figure 5 for the 1 and 7 day(s) old *L. monocytogenes* and *S.* Typhimurium biofilms.

For *L. monocytogenes*, it can be concluded that the total biofilm mass of the untreated biofilm increased (*p* ≤ 0.05) as the biofilm aged, which has been observed before in the research of Govaert et al. (2018a). CAP treatment did not significantly (*p* > 0.05) influence the total biofilm mass (for both biofilm ages), which can be explained by the fact that the applied treatment procedure (see section “CAP Equipment, Hydrogen Peroxide Solution Preparation, and Inactivation Procedures”) cannot result in any removal of the treated biofilm. Following H_2_O_2_ treatment, on the other hand, significantly (*p* ≤ 0.05) lower OD values were obtained compared to the untreated biofilms, and this for both biofilm ages and independent of the applied treatment time and concentration.

For *S.* Typhimurium, no significant (*p* > 0.05) differences in biomass were observed for both biofilm ages, which is again a confirmation of the research of Govaert et al. (2018a). As for *L. monocytogenes*, CAP treatment of both the 1 and 7 day(s) old biofilms did not influence (*p* > 0.05) the total biofilm mass since again no decrease in OD was observed following this treatment. However, similar to the results obtained for *L. monocytogenes*, H_2_O_2_ treatment of the 1 and 7 day(s) old *S.* Typhimurium biofilms resulted in significantly (*p* ≤ 0.05) lower OD values compared to the untreated corresponding biofilms. Nevertheless, this reduction in OD following H_2_O_2_ treatment was not as pronounced as for the *L. monocytogenes* biofilms.

### Remaining Percentage of H_2_O_2_ Following Contact of the H_2_O_2_ Solutions With the Model Biofilms

**FIGURE 6:**
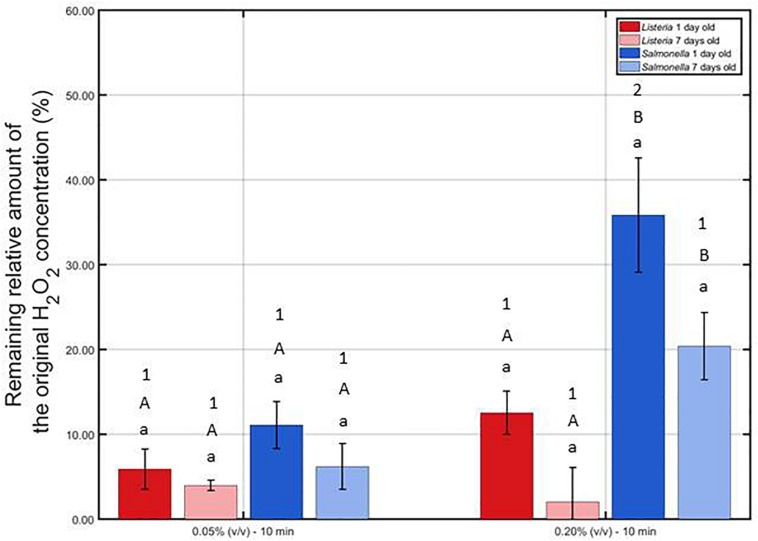
Remaining relative amount of the original H_2_O_2_ solution (%) following contact of the 1 and 7 day(s) old *L. monocytogenes* and *S.* Typhimurium biofilms with the 0.05 and 0.20% (v/v) H_2_O_2_ solutions for 10 min. For each condition, two independent replicates have been performed. Average values and corresponding standard deviations have been calculated and presented in the figure. Separate ANOVA tests were performed to determine the effect of one influencing factor (e.g., biofilm age) on the remaining percentage while all other factors (e.g., H_2_O_2_ concentration and species) were kept constant. Significant (*p* ≤ 0.05) differences have been indicated by means of different lowercase letters (influence biofilm age), uppercase letters (influence species), and numbers (influence H_2_O_2_ concentration), with “a,” “A,” and “1” bearing the lowest value.

The remaining percentage of the initial H_2_O_2_ concentration [i.e., 0.05 or 0.20% (v/v)] has been calculated and presented in Figure 6 for each model biofilm and both examined concentrations.

It can be observed that at the lowest H_2_O_2_ concentration, the remaining relative amounts were not influenced (*p* > 0.05) by the biofilm age and the biofilm forming species. At the highest concentration, on the other hand, an increase in biofilm age resulted in a lower remaining percentage, although not significantly (*p* > 0.05). In addition, for both biofilm ages, the remaining percentage was lower (*p* ≤ 0.05) for *L. monocytogenes* than for the *S.* Typhimurium while applying the highest concentration. Consequently, it can be concluded that the catalase content of the *L. monocytogenes* biofilms was higher (*p* > 0.05) than the catalase content of the *S.* Typhimurium biofilms. Finally, comparing the results obtained at both H_2_O_2_ concentrations, it can be observed that the remaining percentages were in general higher for the 0.20% (v/v) solution, although only significantly (*p* ≤ 0.05) proven for the 1 day old *S.* Typhimurium model biofilms. Based on this, it can be concluded that at the highest H_2_O_2_ concentration, the catalase was less capable to destroy a similar percentage of the initial H_2_O_2_ concentration as for the lowest H_2_O_2_ concentration.

## Discussion

### Efficacy of an Individual CAP and an Individual H_2_O_2_ Treatment for Biofilm Inactivation

Within the presented research, an increased biofilm age resulted in an increased resistance toward CAP treatment. This has been reported before in the research of Govaert et al. (2018a), which examined the resistance of *L. monocytogenes* and *S.* Typhimurium model biofilms toward CAP treatment as function of the biofilm age while applying the same treatment characteristics as within the presented study. For *L. monocytogenes*, this increased resistance at an increased biofilm age can be related to an increase in total biofilm mass (Figure 5), limiting the diffusion of plasma species into the biofilm matrix. For *S.* Typhimurium, on the other hand, no significant (*p* > 0.05) differences in biofilm mass were observed between the 1 and 7 day(s) old model biofilms. Within the study of Govaert et al. (2018a), the increased resistance of the 7 days old *S.* Typhimurium model biofilm was therefore deemed to be a consequence of a collapsing biofilm architecture.

The low antimicrobial activity of H_2_O_2_ for the 1 and 7 day(s) old *L. monocytogenes* biofilms can be a result of the general antimicrobial resistance of biofilms, i.e., the EPS matrix limits the diffusion of antimicrobial agents into the biofilm architecture and protects the biofilm-associated cells against various stresses (Kumar and Anand, 1998). However, as mentioned before, H_2_O_2_ treatment already proved to be successful for inactivation of biofilms formed by some particular species (Christensen et al., 1990; Shikongo-Nambabi et al., 2010). In the research of Shikongo-Nambabi et al. (2010) for example, complete inactivation of mature (72 h old) *V. alginolyticus* biofilms was reported following 1 h of H_2_O_2_ treatment at a concentration of 0.20% (v/v). The log-reduction values obtained within the presented study using the same H_2_O_2_ concentration did not come close to the high log-reductions [>5 log_10_ (CFU/cm^2^)] obtained within this previously mentioned research. Nevertheless, it should be emphasized that this might be a consequence of the difference in (i) biofilm formation procedure (e.g., 72 h of incubation), (ii) treatment procedure (e.g., 30 min vs. 1 h), and, above all, (iii) biofilm forming microbial species. The latter is of particular concern since it has been reported by Bridier et al. (2010) that the biofilm architecture highly depends on the specific microorganism and on the specific microbial strain. Therefore, differences between the biofilm architecture of the studied *L. monocytogenes* biofilms and the architecture of previously mentioned *V. alginolyticus* biofilms could result in a different resistance toward H_2_O_2_ treatment. Another possible explanation for the limited efficacy of H_2_O_2_ for inactivation of the investigated (catalase positive) *L. monocytogenes* biofilms might be the presence of catalase in the biofilm. As mentioned in the research of Stewart et al. (2000), catalase might limit the diffusion of H_2_O_2_ and, consequently, also its biocidal potential. Based on Figure 6, it can indeed be confirmed that the catalase present within the *L. monocytogenes* biofilms was able to reduce up to 95% of the original H_2_O_2_ concentration. Another important factor to take into account is the (partial) removal of the biofilm following H_2_O_2_ treatment. In Figure 5, it was observed that the OD values before and after the examined H_2_O_2_ treatments were significantly (*p* ≤ 0.05) different, i.e., the remaining total biofilm mass was significantly (*p* ≤ 0.05) lower after the treatments. The removed parts of the biofilm were not included anymore during the quantification procedure, but this does not necessarily mean that the removed parts/cells were inactivated. Therefore, the killing potential of the H_2_O_2_ solutions could have been overestimated.

Similar as for the *L. monocytogenes* biofilms, the generally high resistance of the *S.* Typhimurium biofilms toward H_2_O_2_ treatment can be explained based on (i) the general resistance of biofilms (Kumar and Anand, 1998), (ii) the presence of catalase (Figure 6), and (iii) the specific 3-dimensional structure of this model biofilm. For the 7 days old *S.* Typhimurium biofilms in particular, the increased efficacy of the H_2_O_2_ treatment at an increased treatment time (while using the highest H_2_O_2_ concentration) cannot be explained based on differences in OD values (Figure 5) since each individual H_2_O_2_ treatment resulted in a similar reduction in biofilm mass. In addition, although only tested following 10 min of H_2_O_2_ treatment, it was observed that (at the highest H_2_O_2_ concentration) the remaining relative amount of H_2_O_2_ decreased at an increased *S.* Typhimurium biofilm age (Figure 6). Consequently, the increased efficacy of aforementioned H_2_O_2_ treatment cannot be explained based on the difference in catalase present within the 1 and 7 day(s) old *S.* Typhimurium biofilms.

Differences between the resistance of both biofilm forming species toward H_2_O_2_ treatment can be explained based on the level of the cells or the biofilm. On the level of the cells, the difference in resistance can be related to a different ability of the H_2_O_2_ molecules to penetrate into the cells, originating from a difference in Gram type. Although Gram negative cells (such as *S.* Typhimurium) are in comparison to Gram positive cells (such as *L. monocytogenes*) in general more resistant toward antibiotics and certain drugs (Exner et al., 2017), the opposite trend was observed for their resistance toward H_2_O_2_. The thick peptidoglycan layer of the *L. monocytogenes* cells possibly resulted in an inhibited penetration of the H_2_O_2_ molecules into the bacterial cells, which reduced the inactivation efficacy of this antibacterial agent. On the level of the biofilm, the difference in resistance can be related to a difference in (i) the presence of catalase and/or (ii) biofilm architecture. The remaining H_2_O_2_ percentages were in general lower for the *L. monocytogenes* biofilms than for those developed by *S.* Typhimurium, although significant (*p* ≤ 0.05) differences were not always present (Figure 6). The difference in biofilm architecture between both (1 day old) biofilms has been confirmed before by means of confocal laser scanning microscopy. In the study of Govaert et al. (2018b), the 1 day old *L. monocytogenes* biofilm appeared to be more dense (and more homogeneously spread out over the surface) than the corresponding *S.* Typhimurium biofilm. Consequently, this higher density could result in a more limited penetration of the H_2_O_2_ molecules into the biofilm architecture of the former species. CLSM images obtained before and after the combined treatment could be used to confirm this hypothesis.

### Efficacy of the Three Combined CAP and H_2_O_2_ Treatments for Biofilm Inactivation

It has been previously reported that CAP treatment of *P. aeruginosa* biofilms resulted in a more porous biofilm matrix, making the biofilm more susceptible to penetration of chlorhexidine as antimicrobial agent (Gupta et al., 2018). Preliminary Confocal Laser Scanning Microscopy images obtained following CAP treatment of the 1 day old *L. monocytogenes* and *S.* Typhimurium biofilms indicated as well that these specific model biofilms tend to become more porous after CAP treatment (see Supplementary Materials). Therefore, the increased porosity of the biofilms possibly resulted in an increased efficacy of the subsequent H_2_O_2_ treatment. In addition to making the biofilm more porous, the induction of sub-lethally injured cells following CAP treatment of the 1 day old biofilms (Figure 4) could have contributed as well. However, it should be emphasized that this treatment sequence only resulted in synergistic effects if the highest H_2_O_2_ concentration was used. At this concentration, the biofilms were in general less capable to reduce the H_2_O_2_ concentration of the original solution (Figure 6), resulting in a relatively higher amount of H_2_O_2_ that was still present to react with the (injured) biofilm-associated cells. While applying the lowest H_2_O_2_ concentration, no preferred treatment sequence could be observed for the 1 day old biofilms, while for the 7 days old biofilms, it would be advised to treat the biofilms first with H_2_O_2_, then with CAP. For the latter biofilm age, removal of the model biofilms (Figure 5) following H_2_O_2_ treatment possibly contributed to the high overall antimicrobial activity of combined treatment (4) (i.e., first H_2_O_2_, then CAP) as this could have resulted in an increased ability of the plasma species to penetrate into the biofilm matrix. In addition, for the 7 days old *S.* Typhimurium biofilm, the induction of sub-lethally injured cells following the mild H_2_O_2_ treatment (Figure 4) could have contributed as well. The fact that combined treatment (4) only resulted in an increased combined treatment efficacy for the 7 days old biofilms is possibly a consequence of a higher volume of biofilm removal following the initial chemical treatment (Figure 5) and/or possible differences in biofilm architecture between both biofilm ages.

In instances where H_2_O_2_ and CAP were applied simultaneously, the potential for an increased combined treatment efficacy to arise is greatly increased. Reactive species from the plasma are able to directly interact with H_2_O_2_ molecules that were able to penetrate the biofilm matrix, possibly leading to dissociation into shorter lived reactive species with higher oxidation potential (e.g., hydroxyl radicals). Furthermore, UV photons generated by the plasma can induce photolysis of H_2_O_2_, leading to further generation of potent hydroxyl radicals directly *in-situ*. In the case of an atmospheric pressure helium discharge operating with small air admixtures arising from impurities within the background gas and treatment chamber, emissions can span the UV-C, UV-B, and UV-A portions of the spectrum (Ikai et al., 2010). For the plasma conditions used within this study, the excited nitrogen line at 337 nm is the most intense emission (Smet et al., 2017), which is not optimal for H_2_O_2_ photolysis, although similar wavelengths were found to be cable of hydroxyl radical generation (Cataldo, 2014). Within the presented study, the simultaneous treatment often resulted in a decreased combined treatment efficacy, i.e., for the 1 day old *L. monocytogenes* biofilms using both examined H_2_O_2_ concentrations and for the 1 day old *S.* Typhimurium biofilms using the lowest H_2_O_2_ concentration. For the 1 day old *L. monocytogenes* biofilms, an additional experiment has been performed, i.e., the H_2_O_2_ solution was replaced by sterile water during the simultaneous treatment procedure (see Supplementary Materials). This additional experiment indicated that the efficacy of this “simultaneous treatment” was equal to the original simultaneous treatment {CAP + H_2_O_2_ [0.05% (v/v)]}, but lower than the efficacy of the individual CAP treatment. Consequently, one could assume that the penetration of the plasma species into the water (or the hydrogen peroxide solution) was not sufficient. Therefore, the reactive plasma species could not sufficiently reach the biofilm-associated cells to obtain high log-reductions. In addition, the remaining H_2_O_2_ concentration was not high enough (Figure 6) to inactivate the 1 day old biofilm-associated cells. For the 7 days old biofilms, a similar additional experiment was performed (see Supplementary Materials). The efficacy of this simultaneous CAP + H_2_O_2_ treatment was lower than the one reported for the original simultaneous treatment {CAP + H_2_O_2_ [0.05% (v/v)]}, but similar to the efficacy of the individual CAP treatment. Based on this, one could assume that the remaining H_2_O_2_ concentration was still high enough to inactivate the 7 days old biofilm-associated cells and/or to remove parts of the biofilm.

Apart from the hypotheses already formulated to explain the difference in resistance between both biofilm forming species (see section “Efficacy of an Individual CAP and an Individual H_2_O_2_ Treatment for Biofilm Inactivation”), the generally higher resistance of the *L. monocytogenes* biofilms toward the combined treatments can as well be a consequence of a difference in biofilm removal (Figure 5), the higher susceptibility of *S.* Typhimurium biofilm-associated cells to induction of sub-lethal injury (Figure 4), and/or the generally lower remaining H_2_O_2_ concentration following treatment of the *L. monocytogenes* biofilms (Figure 6). Similarly, the increased resistance of the biofilms at an increased biofilm age could be explained based on (i) an increased general resistance toward antimicrobial agents/inactivation methods (Govaert et al., 2018a), (ii) a possible difference in biofilm architecture, and/or (iii) a lower susceptibility of the biofilm-associated cells to induction of sub-lethal injury (Figure 4).

Finally, it is very important to emphasize that, even if no increased efficacy was observed for some combined treatments, log-reduction values up to 5.4 and 5.9 log_10_ (CFU/cm^2^) were obtained for inactivation of the *L. monocytogenes* and the *S.* Typhimurium biofilms, respectively. Consequently, these combined treatments could result in an almost complete inactivation of both biofilm forming species. It would be advised, however, to use the highest H_2_O_2_ concentration and treatment condition (3) (i.e., first CAP, then H_2_O_2_), since these treatment conditions generally resulted in the highest overall log-reduction values. Although H_2_O_2_ is considered as GRAS, this relatively high concentration would require the combined treatment procedure to be followed by a thorough rinsing procedure to remove remaining H_2_O_2_ residues. Additionally, as (partial) removal of the biofilms was observed during each of the combined treatment conditions, biofilm formation on nearby surfaces should be avoided in case these removed biofilm parts would still contain viable biofilm-associated cells.

## Conclusion

Overall, the individual H_2_O_2_ treatment proved to be ineffective for biofilm inactivation, which was caused by the presence of catalase within the different model biofilms. The efficacy of the combined treatment conditions was dependent on the biofilm forming species, the biofilm age, and the applied H_2_O_2_ concentration. The *L. monocytogenes* biofilms were in general more resistant toward the investigated combined treatment conditions than the corresponding *S.* Typhimurium biofilms. Moreover, the biofilms became more resistant at an increased biofilm age. Nevertheless, for both biofilm forming species and both biofilm ages, some combined treatment conditions resulted in an increased efficacy of the combined CAP and H_2_O_2_ treatment. This increased combined treatment efficacy was deemed to be a consequence of the induction of sub-lethal injury, partial removal of the biofilm, and/or an increased porosity of the biofilm matrix following the first individual treatment. For some model biofilms, the simultaneous treatment procedure resulted in a decreased combined treatment efficacy, which was probably the result of (i) a limited diffusion of the plasma species into the H_2_O_2_ solution and (ii) the presence of catalase within the biofilms. It should be emphasized, however, that log-reduction values up to 5.42 ± 0.33 log_10_ (CFU/cm^2^) were obtained if the CAP treatment was followed by the H_2_O_2_ treatment, which means that the mature biofilms were almost completely inactivated. Consequently, the combined treatment of CAP and H_2_O_2_ proved to be a promising method for inactivation of biofilms grown on abiotic food contact surfaces.

In order to fully explain the effect of a combination of CAP and antimicrobial agents (such as H_2_O_2_) on biofilms, more research would be required to (i) examine possible differences in biofilm architecture between the different model biofilms, (ii) determine phenotypical changes as function of the biofilm age, and (iii) unravel the specific inactivation mechanism of CAP and these antimicrobial agents for biofilms.

## Data Availability Statement

The datasets generated for this study are available on request to the corresponding author.

## Author Contributions

MG, CS, and JV: conceptualization and supervision. MG and CS: methodology, validation, and formal analysis. MG and DV: software and investigation. JW and JV: resources. MG: data curation, writing-original draft preparation, visualization, and project administration. MG, CS, DV, JW, and JV: writing-review and editing. JV: funding acquisition.

## Conflict of Interest

The authors declare that the research was conducted in the absence of any commercial or financial relationships that could be construed as a potential conflict of interest.
