# Supplementary material for: Combined Effect of Cold Atmospheric Plasma and Hydrogen Peroxide Treatment on Mature Listeria monocytogenes and Salmonella Typhimurium Biofilms
Source: Front Microbiol. 2019 Nov 20;10:2674. doi: 10.3389/fmicb.2019.02674 (PMC6879557; doi:10.3389/fmicb.2019.02674)
Supplement: Supplementary file 1 [file Table_1.docx]

Table 1 P-values statistical analysis for remaining percentage of H_2_O_2_ following contact of the H_2_O_2_ solutions with the model biofilms

| Influence biofilm age | |
| --- | --- |
| *L.* *monocytogenes* – 0.05% (v/v) | p=0.3870 |
| *L.* *monocytogenes* – 0.20% (v/v) | p=0.0906 |
| *S.* Typhimurium – 0.05% (v/v) | p=0.2157 |
| *S.* Typhimurium – 0.20% (v/v) | p=0.1070 |
| Influence biofilm forming species | |
| 1 day old – 0.05% (v/v) | p=0.1820 |
| 7 days old – 0.05% (v/v) | p=0.3736 |
| 1 day old – 0.20% (v/v) | p=0.0444 |
| 7 days old – 0.20% (v/v) | p=0.0445 |
| Influence hydrogen peroxide concentration | |
| *L.* *monocytogenes* - 1 day old | p=0.1148 |
| *L.* *monocytogenes* - 7 days old | p=0.5721 |
| *S.* Typhimurium - 1 day old | p=0.0405 |
| *S.* Typhimurium - 7 days old | p=0.0523 |
