# Supplementary material for: Combined Effect of Cold Atmospheric Plasma and Hydrogen Peroxide Treatment on Mature Listeria monocytogenes and Salmonella Typhimurium Biofilms
Source: Front Microbiol. 2019 Nov 20;10:2674. doi: 10.3389/fmicb.2019.02674 (PMC6879557; doi:10.3389/fmicb.2019.02674)
Supplement: Supplementary file 2 [file Table_2.docx]

Table 2 Comparison of the log-reductions (log_10_ (CFU/cm²)) obtained following the individual CAP treatment, the simultaneous CAP + H_2_O_2_ (0.05% (v/v)) treatment, and the simultaneous CAP + H_2_O treatment. Results have been shown for the 1 and 7 day(s) old *L. monocytogenes* model biofilms.

| **Treatment conditions** | **Log-reductions (log_10_ (CFU/cm²))**  **non-selective medium** |
| --- | --- |
| *Listeria monocytogenes –* 1 day old | |
| 10’ CAP | 2.72±0.51 |
| 10’ CAP + 10’ H_2_O_2_ (0.05% (v/v)) | 1.25±0.50 |
| 10’ CAP + 10’ H_2_O (0.05% (v/v)) | 1.32±0.39 |
| *Listeria monocytogenes –* 7 days old | |
| 10’ CAP | 0.77±0.89 |
| 10’ CAP + 10’ H_2_O_2_ (0.05% (v/v)) | 2.33±1.02 |
| 10’ CAP + 10’ H_2_O (0.05% (v/v)) | 1.08±0.82 |
