# Supplementary material for: Combined Effect of Cold Atmospheric Plasma and Hydrogen Peroxide Treatment on Mature Listeria monocytogenes and Salmonella Typhimurium Biofilms
Source: Front Microbiol. 2019 Nov 20;10:2674. doi: 10.3389/fmicb.2019.02674 (PMC6879557; doi:10.3389/fmicb.2019.02674)
Supplement: Supplementary file 3 [file Image_1.pdf]

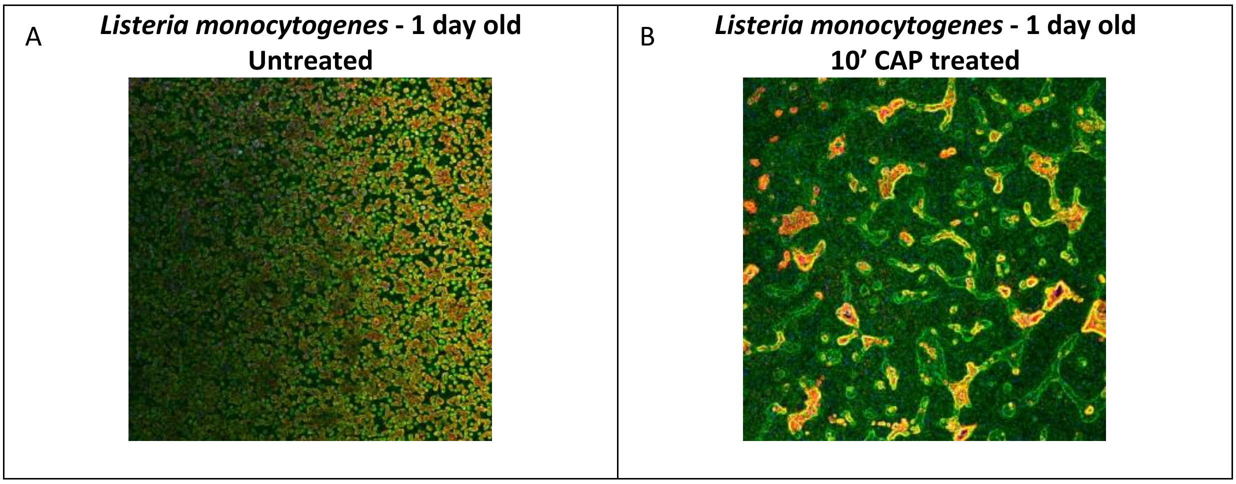

Figure 1 CLSM images obtained (A) before and (B) after CAP treatment of the 1 day old *L. monocytogenes* model biofilm. The (untreated) biofilms were prior to the microscopic analysis stained with Syto 9 (green - healthy cells), Propidium Iodide (red - damaged and/or dead cells), and calcofluor white (blue - polysaccharides in the EPS matrix). Images were taken at different depths of the biofilm, but only the z-stack halfway the total biofilm depth was shown.
